# Are village health sanitation and nutrition committees fulfilling their roles for decentralised health planning and action? A mixed methods study from rural eastern India

**DOI:** 10.1186/s12889-016-2699-4

**Published:** 2016-01-22

**Authors:** Aradhana Srivastava, Rajkumar Gope, Nirmala Nair, Shibanand Rath, Suchitra Rath, Rajesh Sinha, Prabas Sahoo, Pavitra Mohan Biswal, Vijay Singh, Vikash Nath, HPS Sachdev, Jolene Skordis-Worrall, Hassan Haghparast-Bidgoli, Anthony Costello, Audrey Prost, Sanghita Bhattacharyya

**Affiliations:** 1 Public Health Foundation of India, Plot no. 47, Sector 44 Institutional Area, Gurgaon, 122002 New Delhi, Haryana India; 2 Ekjut, Chakradharpur, Jharkhand India; 3 Sitaram Bhartia Institute of Science and Research, New Delhi, India; 4 Institute for Global Health, University College London, 30 Guilford Street, London, WC1N 1EH UK

**Keywords:** Village health sanitation and nutrition committee, Community participation, Decentralized governance, Planning, India

## Abstract

**Background:**

In India, Village Health Sanitation and Nutrition Committees (VHSNCs) are participatory community health forums, but there is little information about their composition, functioning and effectiveness. Our study examined VHSNCs as enablers of participatory action for community health in two rural districts in two states of eastern India – West Singhbhum in Jharkhand and Kendujhar, in Odisha.

**Methods:**

We conducted a cross-sectional survey of 169 VHSNCs and ten qualitative focus group discussions with purposively selected better and poorer performing committees, across the two states. We analysed the quantitative data using descriptive statistics and the qualitative data using a Framework approach.

**Results:**

We found that VHSNCs comprised equitable representation from vulnerable groups when they were formed. More than 75 % members were women. Almost all members belonged to socially disadvantaged classes. Less than 1 % members had received any training. Supervision of committees by district or block officials was rare. Their work focused largely on strengthening village sanitation, conducting health awareness activities, and supporting medical treatment for ill or malnourished children and pregnant mothers. In reality, 62 % committees monitored community health workers, 6.5 % checked sub-centres and 2.4 % monitored drug availability with community health workers. Virtually none monitored data on malnutrition. Community health and nutrition workers acted as conveners and record keepers. Links with the community involved awareness generation and community monitoring of VHSNC activities. Key challenges included irregular meetings, members’ limited understanding of their roles and responsibilities, restrictions on planning and fund utilisation, and weak linkages with the broader health system.

**Conclusions:**

Our study suggests that VHSNCs perform few of their specified functions for decentralized planning and action. If VHSNCs are to be instrumental in improving community health, sanitation and nutrition, they need education, mobilisation and monitoring for formal links with the wider health system.

## Background

Strategies to strengthen community participation in health vary greatly [1]. Many, however, involve communities prioritizing health needs and participating in the design and monitoring of health services [1–5]. After the 1978 Alma Ata Declaration, several countries including Cuba, Tanzania, Zambia, the Philippines and India created village-level health committees to fulfil these roles [2, 6–8].

India has a long history of decentralised governance and involvement of village committees in health planning. The 1946 Bhore Committee report suggested the formation of village health committees to enhance community ‘cooperation’ with health authorities and address local health issues [9]. Village health committees were eventually implemented in India as part of the revival of primary health care in the 1980s, to supervise local health activities with the involvement of community health workers [10].

In 2005 the National Rural Health Mission (NRHM) programme brought sweeping changes to India’s health system, including greater funding and decentralised planning to improve the availability and quality of health services, especially for the rural poor. The NRHM conceptualized the Village Health and Sanitation Committee (VHSC) as responsible for village-level health planning and monitoring, formed within the overall framework of the *Gram Panchayat* (village council), in which women, village members from vulnerable groups and minority communities should be adequately represented [11]. These VHSCs were tasked with preparing village-level health and sanitation improvement plans based on local priorities, and were given an untied fund of Indian Rupees (INR) 10,000 (about US$ 161) per village annually to undertake planned activities. In 2011, VHSCs were renamed ‘Village Health, Sanitation and Nutrition Committees’ (VHSNCs), to expand their role to address nutrition [12]. Committees are now required to maintain data on the nutritional status of women and children, refer severely malnourished children to rehabilitation centres, prepare the nutritional components of the village health plan, and educate community members on nutritional issues [13]. Committees are also asked to supervise Anganwadi Centres (AWCs), which are village-level nutrition and pre-school education centres, and to monitor the Village Health and Nutrition Day (VHND), a monthly event when midwives administer immunisation, antenatal care and provide counselling on recommended maternal and child health practices. [12, 13]. The current strategy envisions VHSNCs as people’s organisations for intersectoral planning and action to address the social determinants of health, and increase people’s utilisation of public health services [14].

Little research exists on these committees, their composition, functioning and effectiveness. Most studies have used only qualitative methods to explore their functioning [15–17], use of funds[18–20] and limitations [21]. One intervention study evaluated a capacity building intervention to strengthen VHSNCs in two districts of Karnataka, southern India [22].

We aimed to understand the current structure and functioning of VHSNCs for decentralised health planning and community action in health, nutrition and sanitation in rural, underserved areas of India. We also looked at factors that affected the functioning of VHSNCs.

## Methods

The study was nested within a cluster-randomised controlled trial testing an intervention involving a community-based worker focused on improving growth in children under two, in two rural districts of eastern India [23]. Strengthening of VHSNCs through a cycle of six quarterly meetings over two years is being conducted in both intervention and control arms as a minimum common service that is sustainable beyond the trial period, with no additional cost to local health services. This data reported in this paper were collected as part of the baseline study of VHSNCs, conducted between December 2013 and February 2014.

### Study population

The study was conducted in the districts of West Singhbhum (Jharkhand) and Kendujhar (Odisha). Both Jharkhand and Odisha are largely rural, with significant tribal populations. Table 1 describes common socio-demographic indicators for these two states. Both are ‘high focus’ states, prioritised by the National Health Mission. The two study districts have a combined population of 3.3 million. Over 80 % of their population lives in rural areas, and female literacy rates in West Singhbhum and Kendujhar are 47 % and 58.7 %, respectively [24]. Over 40 % of residents in both districts are from *Adivasi* (meaning original inhabitant) communities, often referred to as Scheduled Tribes. The two districts are representative of other tribal-dominant districts and high-focus states of India, and findings from these areas may also be relevant to other low and middle-income countries that have sought to support community-based health planning and action in low literacy contexts. The study area includes 120 geographical clusters covering an estimated population of 121,531 across the two districts. Health services at the village level are provided by three community health workers (CHWs):

**Table 1:** Jharkhand and Odisha: demographic and health indicators

Indicators	Odisha	Jharkhand	India
% rural population^a^	83	76	69
% tribal population^a^	23	26	9
Female literacy rate^a^	64	56	66
Infant Mortality Rate^b^	53	38	42
Maternal Mortality Ratio^c^	235	219	178
% institutional deliveries^d^	76	40	73
% children aged 12–23 months fully immunized^d^	60	60	67
% children under five who are stunted^e^	45	50	48
% households covered by improved sanitation facility^e^	16	15	31

^a^Census of India 2011

^b^SRS Bulletin, Oct 2013

^c^SRS estimates 2010–12

^d^Annual Health Survey, 2012–13 (Odisha and Jharkhand); Coverage Evaluation Survey 2009–10 (India)

^e^DLHS-3, 2007–08

- an Anganwadi worker (AWW), who provides supplementary nutrition to pregnant women, lactating mothers and children aged three to six years;
- An Accredited Social Health Activist (ASHA), who helps community members access essential health services and promotes institutional deliveries; and
- An Auxiliary Nurse Midwife (ANM), who provides immunisations, essential medicines and antenatal care.

### Study design

The study, using a mixed methods design, involved two phases of data collection [25]. In the first phase we conducted a cross-sectional quantitative survey of all 169 VHSNCs in the study area (91 in West Singhbhum, Jharkhand, and 78 in Kendujhar, Odisha), in order to obtain information on their composition, training of members, fund availability and activities. We then conducted ten focus group discussions (FGDs) with VHSNC members to gain an in-depth understanding of members’ perception of their roles and functions, levels of participation in planning and activities, and recommendations for improving the committees’ functioning. Qualitative data was collected to explore in-depth the areas covered by quantitative data collection. In the study we first present the quantitative finding and then utilize the qualitative data to analyse the particular aspect in further detail.

**Fig. 1:**
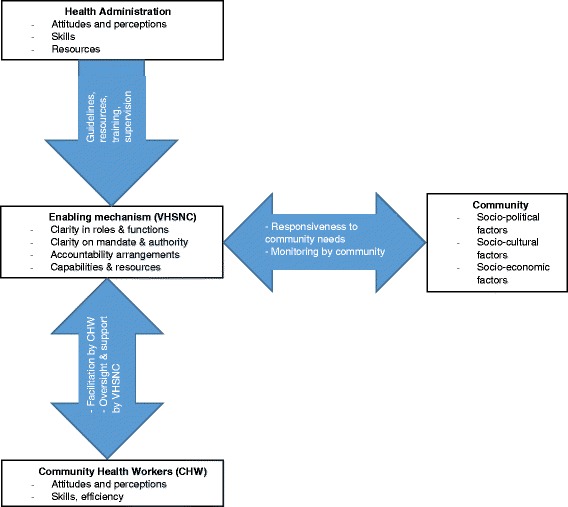
Conceptual Framework for enabling community participation in health through VHSNCs. Note: Adapted from the health governance framework of Brinkerhoff and Bossert (2008)

In order to guide the design of quantitative and qualitative data collection tools, as well as data analysis, we used a conceptual framework for community participation in health and described the VHSNC as an enabling mechanism linking the community, community health workers and the health administration (Fig. 1). Listed with each element are the factors that influence the VHNSCs’ effectiveness. Our conceptual framework was adapted from the health governance framework of Brinkerhoff and Bossert [26] and the framework for the determinants of health facility committee performance offered by McCoy et al. [27]. Four main elements are depicted in relation to the Indian context:

1. The VHSNC is conceptualized as a platform to foster community participation in health. In order for this to happen, members need to be clear about their roles and the committee’s functions, mandate and authority. They also need to have the required capabilities and resources, and must put in place accountability mechanisms.
2. Health administration provides guidelines, resources, capacity building and supportive supervision to the committee. The health administration’s relationship with VHSNCs is influenced by the attitude, perception and skills of staff in the health system, as well as the availability of resources.
3. Community health workers, who are members of the VHSNC, facilitate its functioning, and are in turn monitored and supported by the committee in performing their functions in the village. Their attitudes, perceptions, knowledge and skills also influence their effectiveness.
4. The community, who is the primary beneficiary of the committee’s activities, participates in planning and implementation and also monitors the committee’s performance. The community is also the recipient of mobilisation and awareness generating activities carried out by the VHSNC. Links between the VHSNC and community are influenced by the overall socio-political, cultural and economic context.

Based on a typology of community participation in health programmes, we situated VHSNCs within the ‘community development’ or ‘empowerment’ approach, in which health is defined as a human condition and participation as the planning and management of health activities by the community using professionals as resources and facilitators [28, 29]. Community participation is classified on the basis of how people participate, whether as beneficiaries, in conducting programme activities, in implementing health programmes, in monitoring and evaluating programmes, or in planning and managing health programmes [29].

### Quantitative methods: cross-sectional survey

The purpose of the survey that covered all 169 VHSNCs in the study area was to understand VHNSCs formation and composition, meeting frequency, supervisory mechanisms and activities. Survey respondents were multiple VHSNC members including ASHAs, AWWs, Presidents, Treasurers, Ward members and other members. The study team met with the ASHA and asked her to identify other committee members whom the team then contacted for the survey. All members agreed to participate in the survey. The study field team met with respondents by appointment. They explained the study objectives and purpose of data collection, and sought verbal consent for collecting the data. Data were collected using a structured questionnaire about each VHSNC’s composition, the quantity and type of training received, receipt of supportive supervision, untied fund utilisation, and activities related to maternal, newborn, child and nutrition services in the last 12 months. Consensus answers to all questions were noted in the responses. The questionnaire was translated into the local or indigenous languages spoken in the study areas; Hindi, Oriya and Ho. Information from the survey was also supplemented and verified wherever possible by examining VHSNC records, such as the meeting and expenditure registers. The survey data were entered in Microsoft-Excel. We examined the data for completeness and accuracy using range checks and frequencies, and analysed it using descriptive statistics.

### Qualitative methods

Focus group discussions aimed to gain information about VHSNC’s composition; members’ perception of the committee’s role and responsibilities; CHW’s roles in the committee; the frequency of meetings, attendance and issues discussed; activities conducted; and record keeping systems. Members were also asked about the challenges and facilitators in VHSNC functioning. Using the cross-sectional survey data, we prepared a list of better and comparatively poorer performing VHSNCs based on the regularity of meetings and expenditure of committee funds (better performing committees met with greater regularity and were able to utilize a higher proportion of their funds as compared to poorer performing ones). VHSNCs for the FGDs were selected purposively from this list to include relatively better or poorer functioning ones. Each FGD included between eight and 10 VHSNC members. The details of VHSNC members in the selected committees were already available from the quantitative survey. The study team visited the selected villages, informed all members and invited them for the FGD. The discussion was held once there were eight or more participants. Care was taken to ensure participation of key office bearers, including two community health workers (ASHA and AWW) and the village headman, who was also often the President or Secretary of the committee. We developed semi-structured topic guides with open-ended questions to guide the FGDs, using our conceptual framework and the official guidelines relating to VHSNC structure and functions.

Trained facilitators with knowledge of local languages (Ho and Oriya) carried out the FGDs in village meeting places. Each facilitator was accompanied by a researcher who noted important discussion points and helped re-focus the discussion on to the guide when required. Facilitators explained the objectives of the study, read an informed consent sheet and requested written consent from participants. Facilitator also encouraged all participants to join the discussion, restraining dominant and encouraging quieter participants. Differences in participation across gender and ethnicity were not significant as most groups were homogenous – consisting mostly of women and of tribal and vulnerable social caste groups. Each FGD lasted between 50 and 60 minutes. Discussion of other relevant issues that emerged was also encouraged. At the end of each FGD, the researchers completed a section on notes and observations, intended to capture information on any challenges in conducting the FGD, the ability of respondents to speak freely, and any other relevant observations. All FGDs were recorded using a digital voice recorder and transcribed by a researcher in the local language before translation into English.

The analysis was based on the Framework approach [30]. Three levels of thematic codes were developed and applied to the data. Initially one researcher listed a priori themes based on the FGD guide. Following this, four researchers from the study team jointly identified a set of emerging themes from the transcripts. Finally a third layer of themes was developed, based on synthesis and cross-comparison of data from different groups of respondents in the two study states. Atlas TI software was used to systematically review and code the data. In order to triangulate findings from the quantitative survey and qualitative discussions, we present these results together.

### Ethical approvals

The Institutional Ethics Committee of the Public Health Foundation of India (June 2013, TRC-IEC-163/13), reviewed the protocol and approved the study. An Independent Ethics Committee linked to Ekjut (May 2013), and University College London’s Research Ethics Committee (June 2013, reference 1881/002) also granted ethical approval to the study. All data collection was conducted following informed consent recorded on paper (signed by all participants) after explaining the purpose of the study. Verbal consent was taken from a few participants who were apprehensive about giving anything in writing, though they agreed to participate in the study. Confidentiality of all participants was assured during the data analysis and quotes are given with pseudonyms.

## Results

### Formation and composition of VHNSCs

Most VHSNCs (98 %) in Jharkhand were formed in 2007, and in Odisha, most (81 %) between 2008 and 2010. VHSNCs included an average of nine members in Odisha (Range: 5–12) and an average of 16 (Range: 10–35) in Jharkhand. Table 2 describes the characteristics of VHSNC members by state. Most members were women (just over 75 % in Odisha and 65 % in Jharkhand) and from vulnerable groups - ‘Scheduled Castes, Tribes or Other Backward Castes’, with many more Scheduled Tribe members in Jharkhand. As per Government guidelines, the community health workers (ASHA and AWW) were members of VHSNC in both states, while a Panchayat representative (Ward Member or Pradhan/Mukhiya) was the President. In Jharkhand, ASHAs organised meetings and kept records, while in Odisha record keeping was the role of AWW.

**Table 2:** Characteristic of VHSNC members, by state

		Odisha (*N*= 857)	Jharkhand (*N*=1286)	All (*N*=2143)
Sex	Male (%)	24.7	35.2	31.0
	Female (%)	75.3	64.8	69.0
Social category	Scheduled Tribe (%)	55.1	74.4	47.6
	Scheduled Caste (%)	7.4	6.7	26.0
	Other Backward Class (%)	36.9	18.9	26.1
	Other (%)	0.7	0.0	0.3

Our qualitative data indicated that VHSNCs were formed according to state-level guidelines in both Jharkhand and Odisha.

### Regularity of meetings and attendance

VHSNCs struggled to hold meetings regularly and with full attendance. Six VHSNCs (two in Jharkhand and four in Odisha) of the ten who participated in qualitative discussions reported holding monthly meetings, but faced irregular attendance. It was difficult to agree on a time convenient to all, resulting in either delayed meetings or poor attendance.

Members who could not attend regularly were agricultural or casual labour; finding time for the meeting implied loss of wages. Attendance was especially thin in the harvest season, when members had to work in the fields. Sometimes members migrated out for seasonal labour; that also led to low attendance.

*People want some money when they attend the meeting as they lose their wage for that day*. FGD1, Jharkhand.

Other members not attending meetings regularly included poor and illiterate members and those living in distant hamlets.

*We call people from distant hamlets to attend meetings; but they don’t come. Hence we don’t call them frequently…now we avoid them. We also call youth but they also don’t attend so we take decisions within the smaller group*, FGD5, Jharkhand.

### Linkages with health authorities

#### Training

Guidelines in both states stipulate that VHSNC members should receive a day’s training on their roles and VHSNC functions. Very few members in either Odisha or Jharkhand had received any training related to VHSNC activities in the past 12 months. In Odisha, 13 members out of the 857 who participated in the quantitative survey had received training. In Jharkhand, only two out of 1286 members had received any training on VHSNC roles and responsibilities.

In our FGDs, all members reported receiving some training on their role as VHSNC members or the committee’s functions, though the quality of this training varied widely.

Four VHSNCs in Odisha and two in Jharkhand recalled that they were oriented on their roles and functions as VHSNC members by block-level officials.

*All members got one day of training in the village. During the training we were told to clean the village ponds, roads and bleach wells. And we are doing it through our VHSNC.* FGD2, Odisha

*We were given information on health, diseases among children… we were told that annually we will receive fund…every month we will…discuss on how to prevent diseases.* FGD 5, Jharkhand.

Members noted that ASHAs and AWWs were usually the recipients of detailed training about the VHSNC, while other members, at the most, received only a brief orientation, which many probably did not regard as ‘training’.

Participants in all FGDs expressed the need for further training in order to improve their knowledge and help them perform their roles as VHSNC members more effectively. Areas for desired training spanned members’ roles and functions, management of VHSNCs, planning and financial record keeping to technical knowledge that they could use for awareness building, such as malaria and diarrhoea prevention and food and nutrition.

*We would like to get some training on the role and responsibility of the VHSNC, utilisation of funds and annual planning. We definitely want to know more about VHSNC functions. … The more we know about VHSNC, the more it will help us to improve our village.* FGD1, Odisha.

### Supportive supervision and monitoring

The block and district health administration is required to provide ‘supportive supervision’ to VHSNCs, which implies monitoring their activities as well as helping committees improve their performance by addressing any issues that may occur. According to VHSNC guidelines, district or block level administrators must visit VHSNC meetings at least once every two months to assess their functioning and offer guidance as required. In Odisha, our cross-sectional survey found that only 5.5 % VHSNCs reported any visit by district or block level officials in the previous year to discuss record keeping and expenditure on health activities. In contrast, 96 % VHSNCs in Jharkhand reported visits in the past year by block level officials, most reporting 2–3 visits. Financial audit of VHSNCs by district-level NRHM officials was negligible, though financial reporting to block-level NRHM staff appeared to be robust. In Odisha, 90 % of VHSNCs had reported on their untied fund expenditure, compared with 85 % of VHSNCs in Jharkhand.

In the qualitative discussions, none of the VHSNCs reported any regular supervisory visits by district or block-level officials. In a few instances the ANM, who visited the village weekly for immunisation sessions and VHNDs, also made enquiries about VHSNC functioning.

*District and Block officials never come but the ANM comes to our village two to three times. Sometimes she attends our meetings.* FGD2, Jharkhand

### Linkages with community health workers

#### Oversight and support

The VHSNC is mandated by NHM to provide oversight and support to community-level health services and health workers to ensure smooth functioning and better coordination between them. Table 3 describes the services monitored by VHSNCs. The majority of VHSNCs in Odisha were active in monitoring the AWC, VHND, the mid-day meal scheme (providing meals to primary and upper-primary students in government schools) and services provided by the ASHA and AWW. On the other hand, only 10 % reported monitoring the Sub-Centre and 4 % reported checking drug availability with community health workers. In Jharkhand, most VHSNCs monitored the work of ASHAs, referral of severely acutely malnourished (SAM) children by AWWs, and VHND services including the ANM’s attendance. Only 17 % monitored AWCs and 3 % checked the Sub-Centre. Most of the 'supervision' was informal and no written records were maintained. Differences were statistically significant (*p* < .001) except for the monitoring of Sub-Centres and ANM’s attendance in VHNDs.

**Table 3:** Village health services monitored by VHSNCs

Services monitored	Odisha	Jharkhand	P*
	(*N*= 91)	(*N*=78)	
Anganwadi Centre, *n* (%)	75 (82.4)	13 (16.7)	.000
Sub-Centre, *n* (%)	10 (11.0)	2 (2.6)	.061
Village Health and Nutrition Day, *n* (%)	81 (89.0)	22 (28.2)	.000
ASHA accompanying women for ID, *n* (%)	52 (57.1)	66 (84.6)	.000
Referral of SAM children by AWW, *n* (%)	58 (63.7)	29 (37.2)	.000
Presence of ANM at VHND, *n* (%)	62 (68.1)	43 (55.1)	.057
Presence of drugs with ASHA/ANM/AWW, *n* (%)	4 (4.4)	-	.000
Mid-day meals in school, *n* (%)	68 (74.7)	-	.000
Children reported by VHSNCs for treatment/rehabilitation, (*n*)			
1 child	11	4	
2–3 children	24	3	
More than 3 children	20	1	
VHSNCs with hamlet-wise malnutrition records, *n* (%)	18 (20)	3 (4)	.001
VHSNCs with information on malnourished children, *n* (%)	55 (60)	8 (10)	.000

*P value obtained through Chi-square test

However, the FGDs revealed that members were not regularly monitoring community health services. Reasons included lack of awareness of this role, or trust in the work of the ASHA and AWW. Sometimes male members hesitated to visit the VHND, as only women and children attended them. Only one VHSNC out of the ten where we held FGDs were regularly monitoring the AWC and VHND.

*We never participate in any VHND programme. AWW and ANM have more knowledge than us, so we never interfere in their work. Our ASHA, AWW & ANM have good cooperation between themselves.* FGD5, Odisha.

#### Participation and facilitation by CHWs

Community health workers such as ASHAs and AWWs play very specific roles in the VHSNC. ASHAs convene the VHSNC meetings and maintain records in Jharkhand, while the AWWs perform the same role in Odisha.

*ASHA is the convener. It is her duty to call meetings and maintain register.* FGD4, Jharkhand.

*AWW call the meetings and if someone misses she briefs them about it.* FGD2, Odisha.

#### Record keeping

The VHSNC is mandated to maintain a record of the village population, especially vulnerable groups (pregnant women, children, malnourished children), and events such as births and deaths. ASHA and AWW routinely maintained VHSNC records as well as other records for births, deaths and malnourished children. Record keeping is also essential to maintaining transparency and accountability in VHSNC functioning. In 82 (90 %) VHSNCs in Odisha, AWWs were solely responsible for maintaining VHSNC records, while in 9 (10 %) VHSNCs, AWW and ASHA together maintained records. In Jharkhand, almost all VHSNCs reported having a meeting register maintained by the ASHA. 53 (64 %) VHSNCs in Jharkhand also maintained a separate cashbook to record fund utilisation, and 17 (22 %) VHSNCs maintained a survey register containing village demographic, health and nutrition status information, recorded and updated by ASHAs. However only nine VHSNCs in Odisha and three in Jharkhand carried out a listing of vulnerable families, pregnant women and young children in the last one year.

### Linkages with the community

#### Planning, fund utilisation and mobilisation for health activities

One of the core functions of the VHSNC is to prepare village-level health plans on the basis of local priorities. Table 4 describes the VHNSCs’ activities in relation to planning and the use of untied funds. 83 % of VHSNCs in Odisha reported preparing village health plans, compared with only 8 % in Jharkhand.

**Table 4:** Planning and fund utilisation by VHSNCs

	Odisha *N*=91	Jharkhand *N*=78	P*
VHSNCs that prepared village plan, *n* (%)	76 (83)	6 (8)	.000
VHSNCs that made a list of vulnerable population groups, *n* (%)	9 (10)	3 (4)	.109
VHSNCs that have a bank account, *n* (%)	91 (100)	64 (83)	.000
VHSNCs with bank account that reported expenditure of untied fund in last 12 months, *n* (%)	88 (97)	39 (57)	.000
Amount of untied fund spent (INR) Mean (SD)**	5267.4 (1923.3)	8297.8 (2251.5)	.000^#^
% of total untied fund (INR 10,000/-) spent annually**	53	83	

*P value obtained through Chi-square test

^#^ *P* value obtained through t-test

**For VHSNCs that reported expenditure

However, in practice, few of the VHSNCs who participated in group discussions had ever prepared an annual health plan, though most seemed to be aware of it. Members in six VHSNCs had no knowledge on how to prepare a health plan. They recalled receiving some orientation on preparing plans and formats for recording them, but the process has not been followed. The untied fund was spent according to the fund utilisation guideline provided to them, without any proper plan in place.

*No we do not prepare any plan for utilisation of untied fund. We got a guideline on utilisation of funds, like how much we can spend for bleaching powder, how much to spend for road cleaning, pond cleaning and referral, etc. Accordingly we spend the fund.* FGD3, Odisha

Although our quantitative survey showed that 83 % committees in Odisha made a village health plan, the qualitative findings show that this is not always the case, and that the purpose of the plan was not always understood. Members were either unaware of this responsibility, or complained of a lack of knowledge of the planning process. In one FGD, members blamed community health workers for not facilitating the process.

*The annual plan is not prepared; ASHA, AWW & Ward Member should take a lead on this but they don’t, hence [the planning process] has not been initiated.* FGD1, Odisha.

In another FGD, members complained that they were not able to implement plans due to late arrival of untied funds.

*Whatever plans we develop never work out as untied funds reach late every year; … Government transfers money in the ninth month of the year and asks us to spend it within the next three months, which causes many problems for us.* FGD2, Odisha.

#### Use of untied funds

Each VHSNC is provided with an annual untied fund of INR 10,000 for carrying out community health, nutrition and sanitation-related activities. All VHSNCs must have bank accounts to be able to receive funds allocated to them to carry out their activities. All 91 VHSNCs surveyed in Odisha had a bank account, while 17 % VHSNCs surveyed in Jharkhand were yet to open one. 97 % VHSNCs with bank account in Odisha and 57 % in Jharkhand reported spending their untied fund in the last one year. On average, VHSNCs reporting expenditure in Odisha spent 53 % of their INR 10,000 (USD 160) untied fund last received, while in Jharkhand VHSNCs spent 83 % of their untied funds.

As shown in Table 5, most VHSNCs in both states spent their untied funds on health and sanitation activities (detailed in the qualitative responses discussed below). Only 23 (14 %) VHSNCs reported spending funds on nutrition. The ‘Other’ category had significant amount of expenditure allocated to it, which mainly included spending on meetings, furniture and other items for the VHSNC itself. Mann–Whitney U tests indicated significant difference in mean expenditures between the two districts on health (*p*<.001) and nutrition (*p* = .009), but not in sanitation (*p* = .053).

**Table 5:** Untied fund expenditure by VHSNCs that reported expenditure in the past 12 months

	Odisha		Jharkhand		P*
	% of VHSNCs spending funds *N*=88	Average amount spent (INR) median (Interquartile range)	% of VHSNCs spending funds *N*=37	Average amount spent (INR) median (Interquartile range)	
Health^a^	99	1000 (500)	73	500 (1319)	.000
Nutrition^b^	25	0 (25)	3	0 (0)	.009
Sanitation^c^	93	1000 (200)	89	2350 (3000)	.053
Public Works^d^	5.6	0 (0)	22	0 (0)	.003
Literacy^e^	0	0 (0)	11	0 (0)	--
Other^f^	96.6	2600 (1587)	100	5000 (4500)	.000

* *P* value for the difference in mean amount spent in INR, obtained through the Mann–Whitney U test

Some examples of activities included under the above categories:

^a^Health: Assistance to pregnant mothers for institutional delivery, insecticide spray, organizing health camps and other events for awareness generation

^b^Nutrition: Referring malnourished children to hospital

^c^ Sanitation: Bleaching and paving of wells and tube wells, cleaning village roads and drains

^d^Public Works: Any construction for public infrastructure/civic services, augmenting drinking water supply

^e^Literacy: Assistance to local schools

^f^Others: Meeting expenses, durable and non-durable items for the committee itself, room for committee meetings, display boards, wall painting etc

Our qualitative data indicated that members were aware that they would get an annual fund and knew how it ‘should’ be utilised.

*We were told that ten thousand [INR] will come to our village…. Health fund will be spent for cleaning of drains, referring malnourished children to hospital, preparing banners, display boards and wall writings. Maximum fund will be spent for cleanliness.* FGD5, Jharkhand

Members reported utilising the annual untied VHSNC fund on a wide range of activities related to health, nutrition, village sanitation and hygiene and also to meet the needs of the committee itself. They discussed and took decisions jointly on fund utilisation in meetings.

*Generally in every meeting we discuss health and sanitation in the village, cleaning of the village road, bleaching of tube well, insecticide spray, night assistance to pregnant mothers, supplying drinking water in summer season, organizing health camps, bank passbook update.* FGD2, Odisha

*We have talked about repairing the tube well, cleaning the village and financial assistance to patients. … [We also discussed] care of pregnant mothers and institutional delivery, [the need to] spread awareness about pregnant mothers diet, how to prevent diseases in children.* FGD5, Jharkhand

#### Monitoring by the community

The linkage between a VHSNC and the community is meant to be operationalised through *Gram Sabha*s or village council meetings, in which the community collectively reviews the VHSNC’s performance. We found very little evidence of a formal system of community monitoring of VHSNCs. In our cross-sectional survey, only 22 % of VHSNCs in Odisha and 31 % of VHSNCs in Jharkhand reported the community ever asked about their activities. In our qualitative discussions, all five VHSNCs in Odisha and one in Jharkhand reported sharing the details of untied fund expenditure with the community, when asked. Quantitative data shows that in 30 % VHSNCs in Jharkhand and 22 % in Odisha reported a *Gram Sabha* (village council) meeting being held in their area to review VHSNC functioning.

*Villagers also monitor the progress and utilisation of money. Sometime we present the expenditure details in front of villagers when they require it.* FGD2, Odisha.

#### Mobilizing community members for health activities

One of the responsibilities of VHSNC members is to raise awareness and educate community members on key health, nutrition and sanitation issues within the community. Participants highlighted several activities including counselling women to access antenatal care, advising pregnant women and mothers about diet and nutrition, discouraging alcohol consumption and counselling families on malaria prevention through the use of mosquito nets and keeping surroundings clean.

*Last year, we visited all the households and educated them about the use of mosquito nets and cleaning of surroundings [for malaria prevention].* FGD5, Odisha

Among health activities, VHSNCs focused significantly on helping people to access healthcare. This included helping pregnant women access institutional delivery and supporting elderly and poor patients in accessing healthcare. The committee also assisted patients with specific conditions like tuberculosis and accessing treatment for diarrhoea. Pregnant women and mothers were also counselled on antenatal care and nutrition. Activities for malaria control and environmental hygiene were also significant, and included insecticide spraying and cleaning of roads, waterlogged areas and bushes.

*Once VHSNC members helped [ASHA] take a pregnant lady to hospital at night. They arranged money and vehicle to take her to hospital. The VHSNC donated INR 300.00 to that mother. We also once referred a tuberculosis patient and three malnourished children to hospital through VHSNC fund after discussion with members.* FGD3, Odisha

*VHSNC is getting ten thousand rupees per year and we spend the money for referral of poor people to hospital, treatment of patients, road cleaning, tube well cleaning and also insecticide spray.* FGD2, Jharkhand

With respect to nutrition, the main activity for VHSNCs in both districts was referring severely malnourished children to hospitals. Some members also reported counselling women on nutrition during pregnancy and lactation, and for young children.

*If we identify a child with low weight we register it and refer [to health centre for treatment]. We referred a low-grade [malnourished] baby and its mother to hospital [and] gave [them] INR 200; now both mother and baby are fine. We referred two children with low weight this time.* FGD2, Odisha

Sanitation and hygiene works included cleaning roads, drains and water sources like ponds and wells, and lining them with bleaching powder to prevent infections.

Expenditure on VHSNC’s own requirements included purchasing furniture, utensils, sheets, lights, banners and display boards for the meeting venue, and snacks to be served during meetings.

*We have spent more money on purchasing of some items for committee. We spent on purchasing of polythene [sheet] and lights. Now we have [around] INR 3000 unspent amount and we are planning [to procure] a welcome board and display boards.* FGD3, Jharkhand

## Discussion

While committees have been generally constituted according to the guidelines in both districts, their training and compliance with planning and specified functions was very weak. Clarity in roles and functions, mandate and authority are the prerequisites for an effective VHSNC and greater health system support and supervision enable stronger community health committees [4]. Our study found relatively weak linkages between the VHNSCs and the health system, particularly in Jharkhand. This could be on account of overall weaker local governance in the state. Weak supportive supervision of VHSNCs by the health system was common to both states as reported in other studies [19, 21]. VHSNC members in both states were unclear about their roles, with the exception of the CHWs, who have specific roles in the committees as conveners. This has been observed in other studies in India [15, 16, 18, 20, 31]. Few members received formal training in VHSNC functioning as found by VHSNC members interviewed in a study in Maharashtra [16]. Though socially and geographically marginalised groups were represented in the committees, their participation was irregular. This has equity implications, as frequently absent members were completely excluded from the VHSNC process over time [21].

Our study had strengths and limitations. It covered a large number of VHSNCs in a quantitative survey and also used qualitative methods for an in-depth investigation. Having data from two adjacent districts across two states provides useful lessons to share. However, our findings cannot be generalised to the states in their entirety, or to other parts of India. Our self-reported data in both our quantitative survey and group discussions may have been subject to social desirability bias, as members may have felt pressured to report adhering to government guidelines. In FGDs, group dynamics could have influenced responses as participation was not uniform and some members were more vocal than others. The community health workers had a tendency to project a more positive picture of the committee as its functioning reflected on their effectiveness in coordinating it. We could infer from this that findings relating to any challenges in participation and decision making may have been missed or inadequately reflected. These biases could explain discrepancy between quantitative and qualitative responses for some aspects like monitoring of community health services and making of annual plans by VHSNCs. Lastly, owing to time and resource limitations, the study focused exclusively on the committees themselves, and did not explore the perspectives or the health authorities or community at large regarding these committees, which could have strengthened some of our inferences.

Our comprehensive analysis of all VHSNCs in two districts of India adds to the global evidence on the effectiveness of village-level committees for decentralised health planning and action. Existing evidence on village-level health committees, largely drawn from African studies, shows these structures often suffer from weak community linkages and participation, even when they operate within well-established systems [2, 7, 32]. This is mainly on account of a lack of inclusive membership, poor community engagement and weak support from the health authorities. Having a longstanding tradition of centralised governance is also thought to weaken participatory community-level bodies [8].

Studies highlight the need to ensure inclusive participation and community mobilisation [2, 7]. Strong committees are well supported by district or regional health system, work with relative autonomy, free from political interference and enjoy community confidence [2, 4, 33, 34]. Inadequate support in terms of infrastructure, resources, support and training prevents committees from performing to their potential [32]. Sustained engagement, advocacy and education of committee members and the larger community was suggested to improve role clarity, enhance member skills and activate the committees to perform their functions [8]. These suggestions have strong relevance in India too.

Our quantitative data from questionnaires were often contradicted by focus group discussions. In questionnaires members report that they undertake diverse health-related activities for the community, but the group discussions show that activities focus only on village sanitation works, facilitating treatment of patients and organizing health awareness camps. Most untied funds, are spent on environmental sanitation and ‘other expenses’. Similar lack of focus on mandated activities by VHSNCs has been reported in other studies [20].

Findings reveal that CHWs were strongly linked to the VHSNCs, fulfilling their roles as conveners, facilitators and record keepers of the committees. They were also monitored by the block level health authorities on their performance of this role. However, there were gaps in the monitoring by VHSNCs of community health services provided by CHWs. The VHSNCs’ links with community health workers are guided by the district and state health administration. Better guidance from the health authorities in Odisha led to better linkages of VHSNCs with CHWs, with members more actively involved in Village Health and Nutrition Day and monitoring of the Anganwadi centers. In some committees where monitoring was lax, members expressed full faith in the CHWs. Research evidence from Africa has found that CHWs often dominate committees as they have more knowledge and power compared to other members [35, 36]. In our setting, this was evidenced through the tendency of CHWs to dominate discussions in some of the FGDs. Though not explicitly stated by the members, this could be a possible factor preventing them from monitoring services provided by CHWs. Other studies have also found that CHWs often resent and object to the monitoring by committees [36], but this aspect was not explored in our study.

Community mobilisation and health awareness activities were supported by VHSNCs in both states. However, there was little evidence of any systematic monitoring of VHSNC by the community, with members only mentioning that they shared VHSNC details with the community ‘when asked’. Linkage with the community has been explored in other studies on VHSNCs only from the point of view of participation of Panchayati Raj institutions and not the village community as such [21, 31]. Community participation is heavily influenced by prevalent socio-cultural norms, political and economic relations, as they determine the dynamics of interaction and participation within the community [37]. A recent review of the NHM has suggested that training should be undertaken for VHSNCs at state and district levels, with trainers specializing in social mobilisation. Greater state support is also recommended for village health planning, community monitoring of community health services and local collective action for health promotion. This will enhance the participatory nature of VHSNCs [13].

## Conclusion

VHSNCs offer an opportunity for bottom-up planning in community health. Our study has highlighted comprehensive weaknesses among VHSNCs that must be urgently addressed. Health authorities must train and help VHSNCs through supportive supervision. Members require orientation regarding their roles and responsibilities, technical aspects of health, nutrition and sanitation, and participatory processes related to making plans and using untied funds. Local non-governmental organisations could help with training and encouraging community participation to make them more accountable. VHSNCs need assistance to plan effectively, in order to allocate their funds rationally between health, nutrition and sanitation activities in the community. Well-designed annual health plans could be integrated into block and district-level health plans, thereby strengthening the decentralised health planning process recommended by India’s national guidelines.

## Abbreviations

ANM: auxiliary nurse midwife
ASHA: accredited social health activist
AWC: Anganwadi centre
AWW: Anganwadi worker
CHW: community health worker
FGD: focus group discussion
INR: Indian rupee
NHM: National health mission
NRHM: National rural health mission
SAM: severely acutely malnourished
VHND: Village health and nutrition day
VHSC: Village health and sanitation committee
VHSNC: Village health sanitation and nutrition committee
